# A Sub-Regional Calibration Method That Can Accomplish Error Compensation for Photoelectric Scanning Measurement Network

**DOI:** 10.3390/s19092117

**Published:** 2019-05-07

**Authors:** Zhenyu Zhang, Yongjie Ren, Linghui Yang, Jiarui Lin, Shendong Shi, Jigui Zhu

**Affiliations:** State Key Laboratory of Precision Measuring Technology and Instruments, Tianjin University, Tianjin 300072, China; zhang_zhenyu@tju.edu.cn (Z.Z.); icelinker@tju.edu.cn (L.Y.); linjr@tju.edu.cn (J.L.); ssd2168@tju.edu.cn (S.S.); jiguizhu@tju.edu.cn (J.Z.)

**Keywords:** photoelectric scanning, sub-regional calibration, error compensation, large-scale metrology

## Abstract

In the measurement process of photoelectric scanning measurement network, the laser surface edge area has lower measurement accuracy than the middle area due to the geometrical distortions of the laser surface of the transmitter. This paper presents a sub-regional calibration method that can accomplish error compensation for the measurement system. Unlike the camera sub-regional calibration, the regional division and identification of the laser surface are more difficult. In this paper, the pitch angle in the transmitter coordinate frame of the spatial point was used as the basis for the division and identification of the laser surface. In the calibration process, the laser surface of the transmitter was divided into different regions and each region was calibrated independently, so that an intrinsic parameters database containing the intrinsic parameters of different regions could be established. Based on the database, the region identification and error compensation algorithm were designed, and comparison experiments were carried out. With the novel calibration method, the measurement accuracy of the system had an obvious upgrade, especially at the edges of the laser surface within a certain measurement area, which could enlarge the effective measurement area of the transmitter and would broaden and deepen the application fields of photoelectric scanning measurement network.

## 1. Introduction

Large-scale metrology, as an indispensable role for modern industry, is routinely used in large-scale equipment manufacturing industry and precision engineering. The measurement systems for large-scale metrology can be classified by means of their system topology into centralized and distributed systems [1]. The centralized measurement system is a stand-alone unit that can independently measure spatial coordinates. The common centralized measurement systems include laser trackers [2], laser radars [3], tachymeters, laser scanners [4], and more. Laser scanners and laser radars are laser based range finders which enable a measurement of non-cooperative targets and featureless surfaces. For some laser scanners, the 3D coordinate measurement is based on triangulation. The distributed measurement system consists of several, separate and independent units whose separately gathered measurement information needs to be jointly processed in order for the system to determine the coordinates of a point. It is also possible to use centralized systems in combination as a distributed system, such as laser trackers. Compared with the centralized measurement system, the distributed system has advantages of measurement range scalability, which is very practical in large-scale metrology. There have been several distributed measurement systems provided for large-scale measurement, including theodolite networks [5,6], digital photogrammetry [7] and laser trackers. However, the theodolite networks cannot measure automatically in real time, the digital photogrammetry has a limited field of view and the laser tracker is unable to measure more than one point concurrently. Compared with these previous methods, photoelectric scanning measurement network, having concurrent measurement capability, has developed rapidly and shown great potential in the large-scale manufacturing industry [8]. As a representative, the workshop measurement positioning system (wMPS) has been widely advocated in the manufacturing industry, and intensive research efforts have been conducted on this system [9,10,11,12,13,14].

The wMPS consists of several transmitters, receivers, a signal processor and a terminal computer. As a node of the system, the calibration for intrinsic parameters of the transmitter is a crucial link in the whole measurement process, which can directly affect the final measurement accuracy [15]. However, there are relatively few methods for calibrating the intrinsic parameters of the transmitter. The earliest calibration method was to directly measure the point coordinates in the laser surfaces of the transmitter by using the theodolite measuring system and the white screen with the grid line, and then fitting the laser surface by the least squares method, which was very inefficient and had a large manual error. Zhao et al. [16] designed an automatic calibration method by using the laser tracker and the precise control network, which improved calibration efficiency and reduced manual error. In both existing methods, the laser surfaces were represented by plane equations. In the measurement process, we discovered that the laser surface edge area had lower measurement accuracy than the middle area, which reduced the effective measurement area of the transmitter. There are many factors that cause this phenomenon, the most important one of which is that the laser surface is not an ideal plane. The distortion of a cylindrical lens in a laser module and assembly errors of a component can introduce the geometrical distortions of the laser surface.

In order to reduce the error caused by laser surface distortions, Wang et al. [17] proposed a new laser surface mathematical model. Based on the quadratic interpolation principle, the quadratic surface equation was used to represent the laser surface, and the corresponding calibration and measurement algorithms were designed [17]. This method was computationally intensive, and its improvement in measurement accuracy was limited due to the existence of the Runge’s phenomenon. Therefore, in this paper we used sub-regional interpolation to build the laser surface mathematical model, which could better represent the laser surface and had a smaller amount of calculation. The sub-regional calibration has been proposed for camera calibration in photogrammetry [18], but for the wMPS the sub-regional calibration is innovative, which is different from the camera sub-regional calibration. The camera sub-regional calibration is to divide the imaging plane of the camera into different areas, and respectively calibrate the corresponding camera parameters and distortion parameters. Because the imaging plane is rectangular, its area division and recognition are more direct. The laser surface of the transmitter is a fan-shaped surface, so the division and identification of the surface are more difficult. In this paper, the pitch angle in transmitter coordinate frame of the spatial point was used as the basis for the division and identification of the laser surface.

In the proposed method, a high-accuracy 3-axis turntable and a precise 3-D coordinate control network were introduced. The transmitter for calibration needs be fixed on the turntable so that different regions of the laser surface can scan the spatial fixed control points along with the rotation of the turntable. The key to this process is to establish the corresponding coordinate system of each instrument, which is utilized to obtain the coordinates of the spatial points in the coordinate system of the transmitter under different rotation angles of the turntable. The laser surface can be divided into different regions, and each region will have a group of intrinsic parameters so that we can create a database of intrinsic parameters. In the following measurement process, the region identification arithmetic and error compensation algorithm were designed based on the database of intrinsic parameters. A series experiments were been carried out and confirmed that the novel calibration method can improve the measurement accuracy, especially at the edges of the laser surface. This paper is organized as follows: Section 2 gives a description of the sub-regional calibration mechanism. Section 3 presents the mathematical solution, including the sub-regional calibration algorithm and the error compensation algorithm. The experimental platform is set up, and experiments are prepared in Section 4 to verify the validity of the novel calibration method. Finally, in Section 5, we present concluding remarks and potential future improvements.

## 2. The Principle of Calibration

The novel calibration process includes the establishment of the corresponding coordinate frames and the sub-regional calibration. The detailed calibration process is as follow.

### 2.1. The Establishment of the Corresponding Coordinate Frames

**Figure 1:**
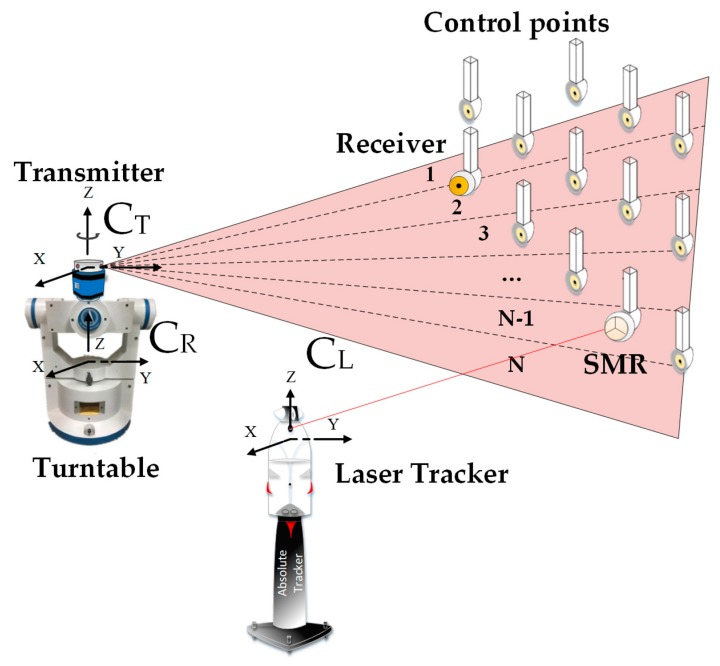
The schematic of the sub-regional calibration.

Establishing the corresponding coordinate frame of each instrument is the key to the novel calibration method. A high-accuracy 3-axis turntable and a precise 3-D coordinate control network are introduced in this method. As shown in Figure 1, there are three coordinate frames introduced, including the turntable coordinate frame, the reference coordinate frame and the transmitter coordinate frame. The three coordinate frames are respectively defined as follows. The precise 3-D coordinate control network is established by the laser tracker, so the coordinate frame of laser tracker can be treated as the reference coordinate frame, which is defined as O_l_-x_l_y_l_z_l_ (C_L_). The turntable coordinate frame O_r_-x_r_y_r_z_r_ (C_R_) is defined as follows. The middle shaft of the turntable is defined as X axis while the inner and outer shafts are treated as YZ axis based on the right-hand rule. The origin O is the rotation center. For the transmitter coordinate frame O_t_-x_t_y_t_z_t_ (C_T_), the rotating shaft of the transmitter is defined as Z axis. The origin O is the intersection of laser plane 1 and Z axis. X axis is in laser plane 1 at the initial time and perpendicular to Z axis. Y axis is determined according to the right-hand rule.

**Figure 2:**
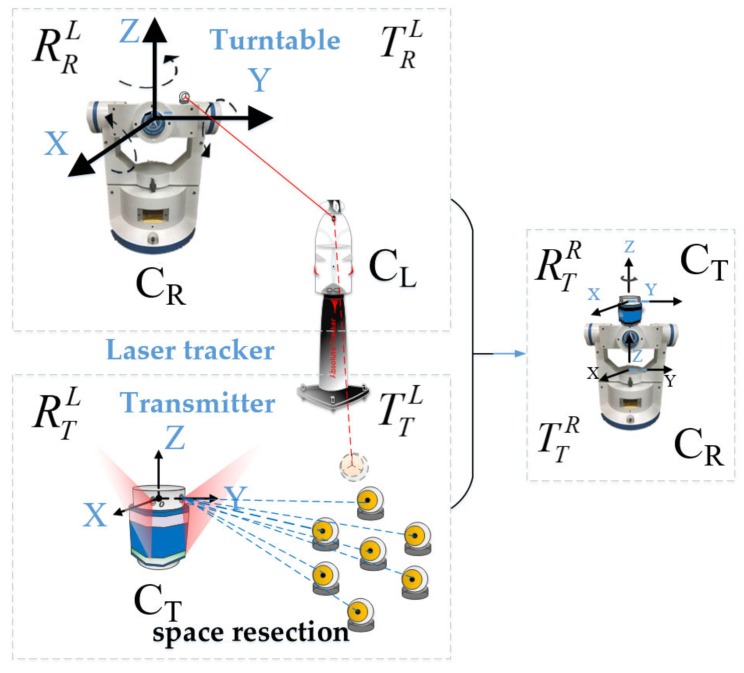
The spatial calibration between the transmitter and the turntable.

Before the sub-regional calibration, a precise calibration with the reference frame O_l_-x_l_y_l_z_l_ (C_L_) is carried out to obtain the spatial relationship between the transmitter and the turntable. As shown in Figure 1, we first need to establish a precise control network with the laser tracker. We then utilize the laser tracker to obtain the spatial relationship between the turntable and the reference coordinate frame based on fitting algorithms. What calls for special attention, is that the position of laser tracker needs to stay fixed during this process. As illustrated in Figure 2, with the help of laser tracker, we can acquire different circular locus’ centers of the three rotation shafts based on circle fitting, and then fit the three direction vectors together based on the collinear constraints and vertical constraints utilizing the least square method. The result of the fitting process can be expressed as a rotation matrix defined as $R_{L}^{R}$ The coordinates of the turntable rotation center can then be obtained by the sphere fitting method, which is defined as $T_{L}^{R}$. Based on transformation principle of coordinate system, we have

(1) $$\{\begin{array}{c} R_{R}^{L}={\left(R_{L}^{R}\right)}^{-1}\\ T_{R}^{L}=-{\left(R_{L}^{R}\right)}^{-1}\cdot T_{L}^{R} \end{array}$$

where $R_{R}^{L}$ and $T_{R}^{L}$ can represent the spatial rotation and translation vector from the turntable coordinate frame O_r_-x_r_y_r_z_r_ (C_R_) to the reference coordinate frame O_l_-x_l_y_l_z_l_ (C_L_).

Still keeping the position of the laser tracker unchanged, we then fixed the transmitter on the turntable. Several spatial points were placed in the space and the coordinates of each point were measured by the laser tracker. For each spatial point, there were two corresponding scanning angles in the transmitter. As illustrated in Figure 2, with the coordinates of spatial fixed points and corresponding scan angles of each point, plane constraint equations could be established. Based on space resection, the spatial relationship between the transmitter coordinate frame and the reference coordinate frame could then be calculated. The rotation matrix and translation vector from the transmitter coordinate frame O_t_-x_t_y_t_z_t_ (C_T_) to the reference frame O_l_-x_l_y_l_z_l_ (C_L_) are defined as $R_{T}^{L}$ and $T_{T}^{L}$. Finally, with the rotation matrixes and translation vectors of the turntable and transmitter with respect to the reference coordinate frame, the rotation matrix and translation vector from the transmitter to the turntable could be obtained, which are defined as $R_{T}^{R}$ and $T_{T}^{R}$ , and we have

(2) $$\left\{ \begin{array}{l} R_{T}^{R}=R_{T}^{L}\cdot {\left(R_{R}^{L}\right)}^{-1}\\ T_{T}^{R}=-R_{T}^{L}\cdot {\left(R_{R}^{L}\right)}^{-1}\cdot T_{R}^{L}+T_{T}^{L} \end{array}\right.$$

### 2.2. The Sub-regional Calibration Mechanism

**Figure 3:**
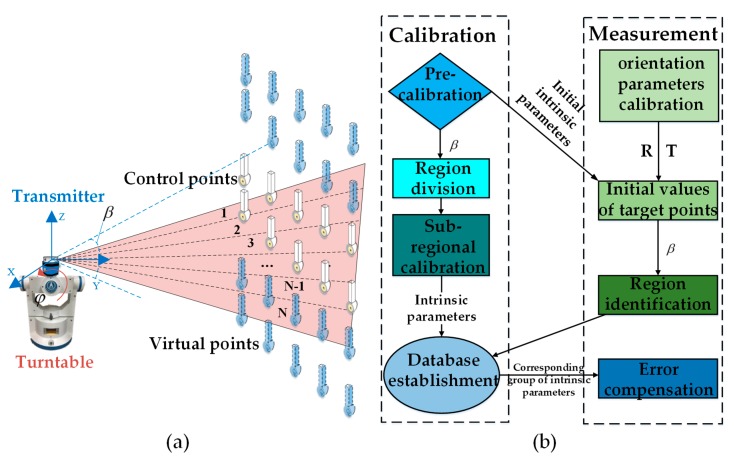
The sub-regional calibration mechanism and the error compensation algorithm. (**a**) The experimental schematic; (**b**) The algorithm structure.

With the corresponding coordinate frames established and the spatial relationship between the transmitter and the turntable acquired, the sub-regional calibration process can be carried out in the follow process. First we need keep the transmitter and the turntable unchanged and utilize the precise control network to calibrate the transmitter preliminarily, which can be called the pre-calibration. For each control point, there will be two corresponding scanning angles. With the coordinate values and corresponding scan angles of the control points, multiple plane constraint equations can be acquired. Based on the space resection, we can acquire a group of intrinsic parameters. We define this group of data as the initial intrinsic parameters, which will be utilized for the region identification of target points. Then with the help of the turntable, we begin to execute the formal calibration. With the rotation of the turntable, we can ensure that the whole laser surface can scan the control network. Based on the theory of relative motion, the clockwise rotation of the turntable along the X axis can be imagined as a counterclockwise rotation of the control network along the X axis of the turntable. As shown in Figure 3a, one control point can be transformed into different virtual points due to the change in rotation angle. With the spatial relationship between the transmitter and the reference frame and the coordinates of control points under the reference frame, we can acquire the pitch angle of the virtual points in the transmitter coordinate frame, which will be the standard of region division. For each virtual point, there will be two corresponding scanning angles. In each region, the coordinates of virtual points in the reference coordinate frame and the corresponding scanning angles can be converted to the plane constraint equations. Based on space resection, the intrinsic parameters in corresponding region can be calculated with the L-M optimization algorithm [17]. In this way, we can build a database of intrinsic parameters, which will be utilized in the error compensation process.

Then in the following measurement process, the initial intrinsic parameters are first utilized to acquire the pitch angle of the target point, which is the evidence of region identification. Based on forward intersection, the laser surfaces that have scanned the target point can establish the multiple constraints and we can then calculate the initial coordinates of target point, which can help us acquire the pitch angle of the target point. We can then carry out the error compensation based on the database of intrinsic parameters we have established. The pitch angle of the target point can decide which one of those groups of intrinsic parameters will be selected for error compensation. Finally utilizing the corresponding intrinsic parameters, we acquire a more accurate set of coordinate values for the target point based on forward intersection. The whole process of the sub-regional calibration and the error compensation is explicated in the Figure 3b.

## 3. The Mathematical Solution

### 3.1. The Sub-regional Calibration Algorithm

First we need calculate the initial intrinsic parameters, which is called the pre-calibration. Suppose there are **M** points in the control network. Based on space resection, utilizing the precise control network we can set up multi-plane constraints as follows:

(3) $$\begin{array}{cc} d_{\mathrm{ij}}={\left\|\left(\begin{array}{cccc} a_{i0} & b_{i0} & c_{i0} & d_{i0} \end{array}\right)\cdot \left(\begin{array}{cc} R^{T}(\theta_{i}) & 0\\ 0 & 1 \end{array}\right)\left(\begin{array}{cc} R_{0} & T_{0}\\ 0 & 1 \end{array}\right)\cdot \left(\begin{array}{c} x_{j}\\ y_{j}\\ z_{j}\\ 1 \end{array}\right)\right\|}_{2} & \left(\begin{array}{cc} i=1,2 & 1\leq j\leq M \end{array}\right) \end{array}$$

The **i** is the index of the laser surface of the transmitter, the $R_{0}$ and $T_{0}$ is the initial orientation parameters of the transmitter in the reference coordinate frame. The $R^{T}(\theta_{i})$ is the rotation matrix determined by the scanning angle $\theta_{i}$. The $R_{0}$ and $T_{0}$ can be expressed as **r_01_**, **r_02_**, **r_03_**, **r_04_**, **r_05_**, **r_06_**, **r_07_**, **r_08_**, **r_09_**, **t_x0_**, **t_y0_**, **t_z0_** and based on properties of the rotation identity matrix we have the equations below:

(4) $$\{\begin{array}{c} f_{1}=r_{01}^{2}+r_{02}^{2}+r_{03}^{2}-1=0\\ f_{2}=r_{04}^{2}+r_{05}^{2}+r_{06}^{2}-1=0\\ f_{3}=r_{07}^{2}+r_{08}^{2}+r_{09}^{2}-1=0\\ f_{4}=r_{01}r_{04}+r_{02}r_{05}+r_{03}r_{06}=0\\ f_{5}=r_{01}r_{07}+r_{02}r_{08}+r_{03}r_{09}=0\\ f_{6}=r_{04}r_{07}+r_{05}r_{08}+r_{06}r_{09}=0 \end{array}$$

With Equations (3) and (4), we introduce 14 unknown coefficients, including the initial intrinsic parameters and orientation parameters of the transmitter, while 2**M** + 6 constraints are also introduced. By means of the Lagrange multiplier approach, the objective function can be written as:

(5) $$F=\sum_{i=1}^{2}\sum_{j=1}^{M}\left[{\left(d_{ij}\right)}^{2}\right]+\lambda \cdot \sum_{i=1}^{6}f_{i}^{2}$$

Utilizing the **L-M** algorithm we can acquire the initial intrinsic parameters, which are expressed as **a_10_**, **b_10_**, **c_10_**, **d_10_**, **a_20_**, **b_20_**, **c_20_**, **d_20_** [16].

We then start the formal calibration process. According to sub-regional calibration mechanism, one control point can be transformed to different virtual points due to the change in rotation angle. With the spatial relationship between the transmitter and the reference frame, we can acquire the coordinates of virtual points in the transmitter coordinate frame by the equation bellows:

(6) $$X_{T}=R_{T}^{R}\cdot R_{X}\cdot (R_{R}^{L}\cdot X_{L}+T_{R}^{L})+T_{T}^{R}$$

X_T_ ${\left(\begin{array}{ccc} x_{t} & y_{t} & z_{t} \end{array}\right)}^{T}$ is the coordinates of virtual point in the transmitter coordinate frame and X_L_ ${\left(\begin{array}{ccc} x_{l} & y_{l} & z_{l} \end{array}\right)}^{T}$ is the coordinates of the corresponding control point in the reference coordinate frame. The $\left(\begin{array}{cc} R_{T}^{R} & T_{T}^{R} \end{array}\right)$, $\left(\begin{array}{cc} R_{R}^{L} & T_{R}^{L} \end{array}\right)$ can be obtained in the process of establishing the corresponding coordinate frames and the $R_{X}$ is the clockwise rotation matrix along the X axis of the turntable.

With the coordinates of the virtual point X_T_ ${\left(\begin{array}{ccc} x_{t} & y_{t} & z_{t} \end{array}\right)}^{T}$, we can calculate its pitch angle in the corresponding transmitter coordinate frame, which is defined as β and the equation is as follows:

(7) $$\beta =\arctan(\frac{z_{t}}{\sqrt{x_{t}^{2}+y_{t}^{2}}})$$

The regional division of the laser surface is as follows. The fan angle ϕ of the laser surface, the angle φ between the laser surface and the axis of the transmitter are structural parameters during the manufacture of the transmitter, which are known during the calibration process. Based on the minimum angle principle, we have

(8) cosϕ=cosφ⋅cosγ

where the γ is the pitch angle range of laser surface in the transmitter coordinate frame, supposing that the laser surface is divided into **n** regions. The $\frac{\gamma }{n}$ will be the angle range of each region. Based on the pitch angle β and the angle range of each region, the virtual points can be divided into different regions.

After the region division, we can make an independent calibration in each region with the corresponding virtual points. Take one of the regions as an example. Suppose there are **M** virtual points in this region based on the standard of region division. The coordinates of virtual point X_V_ ${\left(\begin{array}{ccc} x_{vj} & y_{vj} & z_{vj} \end{array}\right)}^{T}$ in the turntable coordinate frame can be acquired as follows:

(9) $$X_{V}=R_{X}\cdot (R_{R}^{L}\cdot X_{L}+T_{R}^{L})$$

For each virtual point, there will be two corresponding scanning angles (defined as $\theta_{1}$ and $\theta_{2}$). The coordinates of the virtual point in the reference coordinate frame and the corresponding scanning angles can be converted to the plane constraint equations, which is described as follows:

(10) $$d_{ij}={\left\|{\left(\begin{array}{c} a_{i}\cos\theta_{i}+b_{i}\sin\theta_{i}\\ a_{i}\sin\theta_{i}-b_{i}\cos\theta_{i}\\ c_{i}\\ d_{i} \end{array}\right)}^{T}\cdot \left(\begin{array}{cc} R & T\\ 0 & 1 \end{array}\right)\cdot \left(\begin{array}{c} x_{vj}\\ y_{vj}\\ z_{vj}\\ 1 \end{array}\right)\right\|}_{2}(\begin{array}{cc} i=1,2 & 1\leq j\leq M \end{array})$$

where the **i** is the index of the scanning optical surface of the transmitter, and the **a_i_**, **b_i_**, **c_i_**, **d_i_** are the intrinsic parameters of the laser surface in this region. The $\theta_{i}$ is the rotation angle of the laser surface. The R and T are the orientation parameters of the transmitter in the turntable coordinate frame, which can be expressed as **r1**, **r2**, **r3**, **r4**, **r5**, **r6**, **r7**, **r8**, **r9**, **tx**, **ty**, **tz** and they also follow the Equation (4).

In each region there are eight unknown coefficients for the intrinsic parameters of two laser surfaces. Considering the orientation parameters of the transmitter, there are a total of 14 unknown coefficients in this optimization. There also are 2**M** + 6 constraints introduced. By means of the Lagrange multiplier approach, the objective function can be written as:

(11) $$F=\sum_{i=1}^{2}\sum_{j=1}^{M}\left[{\left(d_{ij}\right)}^{2}\right]+\lambda \cdot \sum_{i=1}^{6}f_{i}^{2}$$

This group of intrinsic parameters can then be calculated with the **L-M** optimization algorithm. The calibrations for the other regions are the same. After the whole regions have been calibrated, a database of intrinsic parameters has been established.

### 3.2. The Error Compensation Algorithm

In the measurement process, we first need to acquire the orientation parameters of each transmitter written as $R_{t}$ and $T_{t}$, which is called the establishment of the measurement network [19]. Then, with the initial intrinsic parameters and the orientation parameters of each transmitter, we can acquire the pitch angle of the target point. Based on forward intersection, the laser surfaces that have scanned the target point can establish the constraint equation as follows:

(12) $$\begin{array}{cc} (\begin{array}{cccc} a_{i0} & b_{i0} & c_{i0} & d_{i0} \end{array})\cdot \left(\begin{array}{cc} R^{T}(\theta_{i}) & 0\\ 0 & 1 \end{array}\right)\cdot \left(\begin{array}{cc} R_{tj} & T_{tj}\\ 0 & 1 \end{array}\right)\cdot \left(\begin{array}{c} x\\ y\\ z \end{array}\right)=0 & \left(\begin{array}{cc} i=1,2 & 1\leq j\leq N \end{array}\right) \end{array}$$

where the **a_i0_**, **b_i0_**, **c_i0_**, **d_i0_** are the initial intrinsic parameters, and the **i** is the index of the laser surface of the transmitter. The $R^{T}(\theta_{i})$ is the rotation matrix determined by the scanning angle $\theta_{i}$. The $R_{tj}$ and $T_{tj}$ are the orientation parameters of the transmitter. The **j** is the index of the transmitter, supposing there are **N** transmitters in the measurement network. The **x**, **y**, **z** are the initial coordinates of the target point. So, there are a total of 2**N** equations and only 3 unknown coefficients. With the Equation (12), we can acquire the initial coordinates of the target point and then we can calculate the pitch angle of the target point in each transmitter coordinate frame as follows:

(13) $$\{\begin{array}{c} {\left(\begin{array}{ccc} x_{t0} & y_{t0} & z_{t0} \end{array}\right)}^{T}=R_{tj}\cdot {\left(\begin{array}{ccc} x & y & z \end{array}\right)}^{T}+T_{tj}\\ \beta =\arctan(\frac{z_{t0}}{\sqrt{x_{t0}{}^{2}+y_{t0}{}^{2}}}) \end{array}$$

As we know, the β is the evidence of region identification. After region identification, we can carry out the error compensation based on the database of intrinsic parameters. In the error compensation process, we use the corresponding group of intrinsic parameters instead of the initial coordinates of the target point. Based on forward intersection we can then acquire new coordinates of the target point with the constraint equation as follows

(14) $$(\begin{array}{cccc} a_{ci} & b_{ci} & c_{ci} & d_{ci} \end{array})\cdot \left(\begin{array}{cc} R^{T}(\theta_{i}) & 0\\ 0 & 1 \end{array}\right)\cdot \left(\begin{array}{cc} R_{tj} & T_{tj}\\ 0 & 1 \end{array}\right)\cdot X=0$$

where the **a_ci_**, **b_ci_**, **c_ci_**, **d_ci_** are the corresponding group of intrinsic parameters from the database. The $R^{T}(\theta_{i})$ is the rotation matrix determined by the scanning angle $\theta_{i}$. The $R_{tj}$ and $T_{tj}$ are the orientation parameters of the transmitter. There are a total of 2**N** equations and only 3 unknown coefficients as before. Finally, we can calculate the coordinates of the target point written as **X_new_** $\left(\begin{array}{ccc} x^{\prime } & y^{\prime } & z^{\prime } \end{array}\right)$.

## 4. Experiments

### 4.1. Set-Up of the Verification Platform

**Figure 4:**
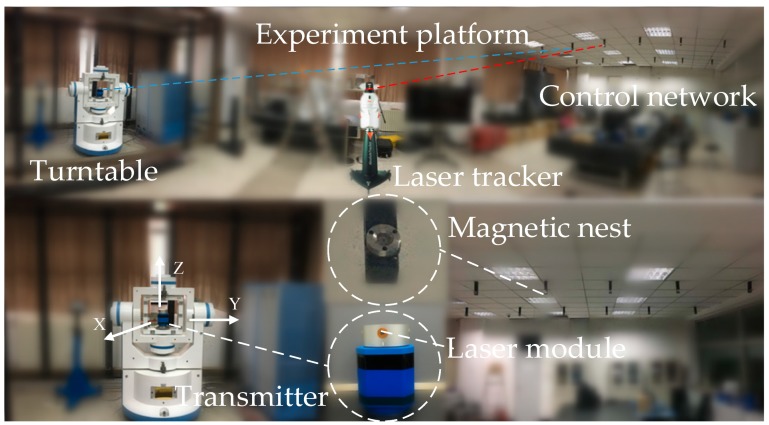
The experiment platform for the novel calibration method.

A high-accuracy 3-axis turntable and a precise 3-D coordinate control network were used to carry out the novel calibration method. The angle position accuracy of 3-axis turntable is within 1″ along each rotation axis. The control network was established by the Leica AT901-LR laser tracker, whose measurement accuracy is 15 μm + 6 μm/m. The transmitter was designed and manufactured by Tianjin University, China. The angle measurement accuracy of the transmitter is 2.6″ (based on three standard deviations) [20]. The platform for calibration using the Leica AT901-LR laser tracker and the 3-axis turntable is shown in Figure 4.

### 4.2. Verification of the Shaft Rotation Stability

**Figure 5:**
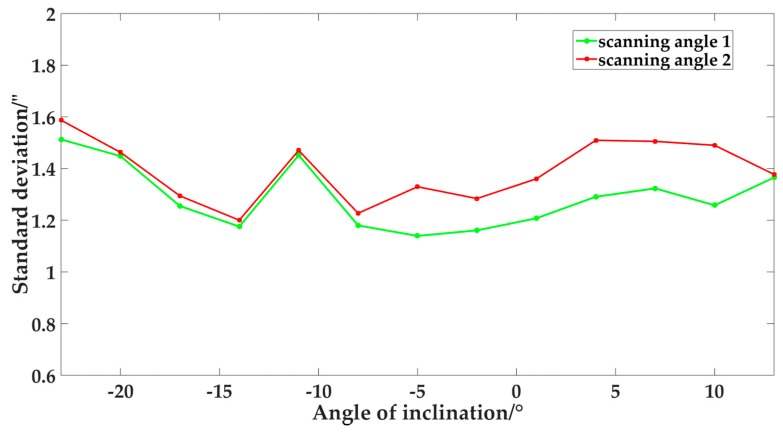
The results of the angle-measurement performance tests.

The wMPS is a type of distributed measurement system based on the principle of angle intersection, so the angle-measurement performance of the single transmitter is the core factor of this system, which is evaluated by the angle-measurement repeatability error. Before the formal calibration, we need verify the stability of angle-measurement performance of the transmitter with the change of transmitter posture. With the help of the turntable, we tested the angle-measurement performance of the transmitter at different inclination angles respectively, and the results of the comparison tests are shown in Figure 5. The ranges of the measurement repeatability error of the scanning angles 1 and 2 were respectively 0.38″ and 0.39″, which indicated that within a certain range, the angle-measurement performance of the transmitter had good stability with the change of inclination angle.

### 4.3. The Sub-Regional Calibration

After the verification platform had been set up, a precise calibration was first carried out to obtain the spatial relationship between the transmitter and the turntable. Then, the sub-regional calibration was carried out and as a contrast we also utilized the conventional calibration method [21] to calibrate the intrinsic parameters of the transmitter. Based on the measurement principle of the wMPS, we need calibrate at least two transmitters to form an effective measurement network. As shown in the Table 1, the intrinsic parameters of the two transmitters were acquired by utilizing the conditional method, which could also be treated as the initial intrinsic parameters of the sub-regional calibration.

Then, utilizing the sub-regional calibration, we established the databases of intrinsic parameters for the two transmitters, which are shown in the Table 2 and Table 3 respectively.

### 4.4. Verification of the Error Compensation

**Figure 6:**
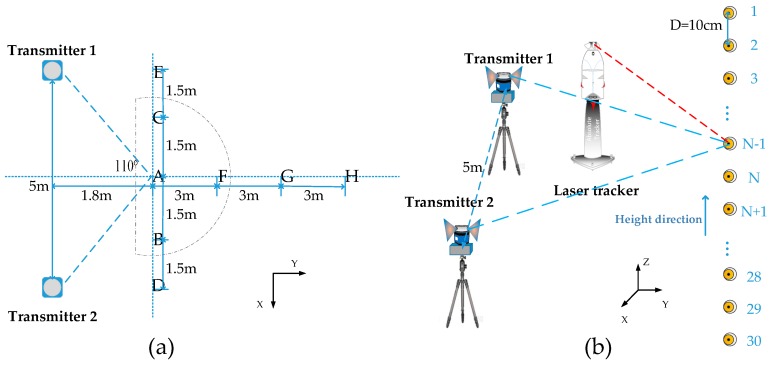
The schematic of the comparison experiments of coordinate measurement. (**a**) The space layout; (**b**) The distribution of test points in each group.

To verify the viability and effectiveness of the proposed error compensation algorithm, a series of comparison experiments were designed to compare the measurement precision between the conventional calibration method and the novel calibration method. The Leica AT901-LR laser tracker, whose accuracy is 15 μm + 6 μm/m, was required to supply standard coordinates. Two transmitters with a distance of 5 m from each other were set up to form a measurement network. As shown in the Figure 6a, we set up 8 sets of comparison experiments in the eight areas A, B, C, D, E, F, G, H. Based on the theory of optimal measurement area proposed by Xiong et al. [22], the intersection angle of the system with the best measurement effect was 110 degrees, that is, the A area. The measurement distances of the B, C, D and E areas were close to those of the A area, but the intersection angles of them gradually changed. The measurement distances of the F, G, and H areas were gradually increased relative to the A area, and were utilized to explore the compensation effect of the novel calibration method under the different measurement distances. As shown in Figure 6b, in each area, we set up a test point every 100 mm in height direction. There were a total of 30 test points for each set of experiments, which were numbered inclusively from 1 through 30. Each point was measured by the wMPS and the laser tracker, respectively. The measured results of the wMPS were resolved twice: the proposed error compensation algorithm and the conventional algorithm. The coordinates measured by the wMPS were translated into the laser tracker coordinate system and then directly compared with the measured results of the laser tracker.

**Figure 7:**
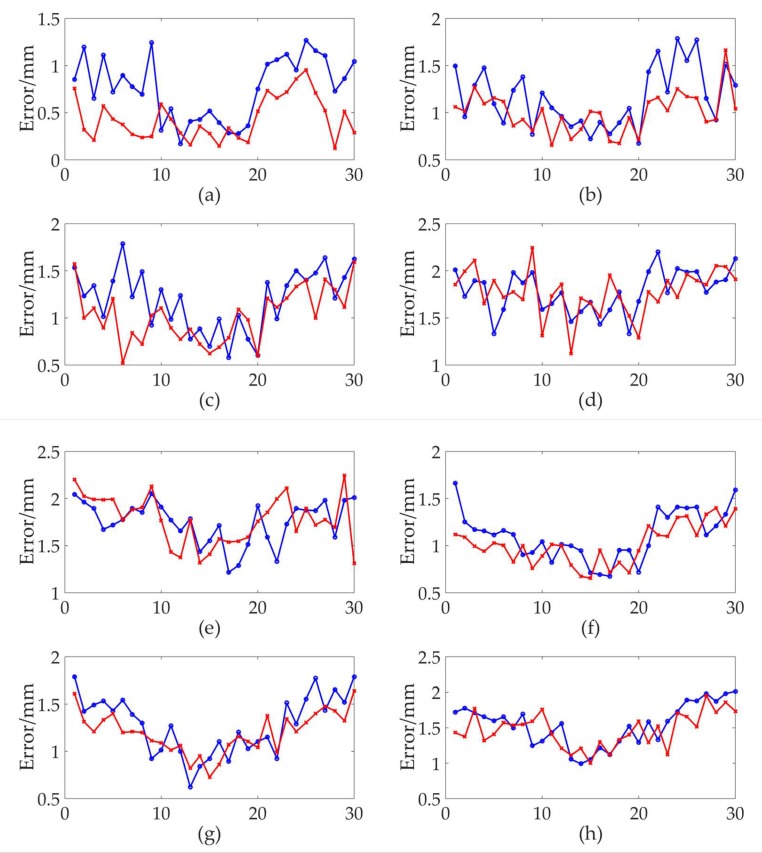
The measurement errors of the two calibration methods (the blue line represents the conventional method and the red line represents the novel method). (**a**) The experimental results of A area; (**b**) The experimental results of B area; (**c**) The experimental results of C area; (**d**) The experimental results of D area; (**e**) The experimental results of E area; (**f**) The experimental results of F area; (**g**) The experimental results of G area; (**h**) The experimental results of H area.

Figure 7 shows the results of the 8 sets of comparison experiments. To reject the possibility of random error appearance, we chose the average error of each region as the evaluation index of measurement performance. Some experimental results can not be seen directly from the graph, but can be obtained through data analysis. Figure 7a–h are the corresponding experimental results for areas A to H, respectively. As can be seen from the Figure 7a, in the A area for the both calibration methods, the measurement errors of the points at the edges of the laser surface were larger than those points at the central areas. The error compensation algorithm of the novel method could reduce the measurement errors of the wMPS, especially for those points at the edges of the laser surface. From point 1 to point 10, the novel calibration method reduced the average error from 0.85 mm to 0.40 mm and from point 21 to point 30 the average error was reduced from 1.01 mm to 0.60 mm. Meanwhile, points from 11 to 20, which were at the central areas of the laser surface, had a little decrease of average error from 0.41 mm to 0.3 0mm. At the points 10 and 17, the measurement errors of the novel method were larger than those of the conventional method. The reason may be that for the sub-regional interpolation algorithm, the interpolation errors at the intersection of different regions are larger, which may be far from the true value.

As shown in Figure 7b,c, compared with the A area, the global measurement errors of the two methods in the B and C areas were larger, which was caused by the change of the intersection angle. Same as the A area, the measurement errors of points at the edges of the laser surface were still larger than those points at the central areas in this two areas. The error compensation algorithm of the novel method could reduce the measurement errors of the wMPS, but the effect was not as obvious as the A area. In the B area, from point 1 to point 10, the novel calibration method reduced the average error from 1.18 mm to 1.04 mm and from point 21 to point 30 the average error was reduced from 1.43 mm to 1.14 mm. The points from 11 to 20 had a little decrease of average error from 0.88 mm to 0.82 mm. In the C area, the average error of the points from 1 to 10 was reduced from 1.32 mm to 1.00 mm and from point 21 to point 30 the average error was reduced from 1.40 mm to 1.27 mm. The points from 11 to 20 had a little decrease of average error from 0.85 mm to 0.80 mm. As shown in Figure 7d,e, in the D and E areas, the measurement results of the wMPS in the two areas were very poor, no matter which calibration method was used. The measurement errors of the points at the edges of the laser surface were also larger than those points at the central areas, but the error compensation algorithm of the novel method had no significant improvement over the conventional method.

We can see from Figure 7a,f–h that as the measurement distance increased, the global measurement error increased gradually, and the measurement errors of the points at the edges of the laser surface were larger than those points at the central areas in these three areas. In the three areas A, F, G, the error compensation algorithm of the novel method could reduce the measurement errors of the wMPS, but the error compensation effect deteriorated as the measurement distance increased. In the F area, from point 1 to point 10, the novel calibration method reduced the average error from 1.15 mm to 0.97 mm, and from point 21 to point 30 the average error was reduced from 1.32 mm to 1.25 mm, and from point 11 to point 20 the average error reduced from 0.85 mm to 0.83 mm, which is very inconspicuous. In the G area, from point 1 to point 10, the novel calibration method reduced the average error from 1.38 mm to 1.27 mm and the points from 21 to 30 had a decrease of average error from 1.37 mm to 1.35 mm. From point 11 to point 20 the average error was reduced from 1.00 mm to 0.98 mm, which was also not obvious. In the H area, the measurement errors of the two calibration methods were close, and the novel method had not improved significantly.

From the above 8 sets of experiments, in the A, B, C, F, and G areas of Figure 7a, the novel calibration method could achieve error compensation, but in the D, E, and H areas, there was no improvement in measurement accuracy compared with the conventional method. In the A, B, C, F, and G areas, the A area had the best error compensation effect, whose intersection angle was the best in the measurement network. The error compensation effect was worse in the B and C areas than in the A area. It is possible that the optical signal recognition error of the receiver photosensitive unit was increased due to the change of the intersection angle, thereby affecting the error compensation effect. The F and G areas were the areas with the worst error compensation, but there was still a certain compensation effect. Moreover, the error compensation effect of the F area was better than that of the G area. There were two factors that affected the final error compensation effect in the two areas: the increase of the optical signal recognition error of the receiver, and the decrease of the length measurement accuracy of the transmitter due to the increase of the measurement distance. In the D, E, and H areas, the novel calibration method had no advantage over the conventional method. This might be due to the fact that the measurement error introduced by the laser surface distortion was not the main error in the areas with poor intersection angle or the far measurement areas. Other error sources such as the transmitter’s own angle measurement error, the receiver optical signal recognition error, the disturbance of air, the uneven refractive index of air, etc., might have a greater impact on the experimental results, which required further experimental exploration. In summary, the novel calibration method has an error compensation effect in the area where the intersection angle is close to the optimal junction angle and the measurement distance is not far, and the error compensation effect is better in the area closer to the optimal measurement area.

The results from the experiment verified the viability and effectiveness of the error compensation algorithm and also indicated that the novel calibration method could improve the measurement accuracy of the wMPS, especially at an edge of the laser surface within a certain measurement area, which would broaden and deepen the fields of the wMPS application.

## 5. Conclusions

A novel intrinsic parameters calibration method that can accomplish error compensation for photoelectric scanning measurement network has been presented. This method uses the sub-regional calibration to establish the intrinsic parameters database and then resolves the target values with error compensation based on the database. This calibration method introduces a high-accuracy 3-axis turntable and a precise 3-D coordinate control network. The calibration principle is expounded in detail, including the establishment of the corresponding coordinate frames and the sub-regional calibration. Moreover, the sub-regional calibration algorithm and error compensation algorithm are also discussed in this paper. The viability and effectiveness of the novel calibration method are verified through the comparison experiments of coordinate measurement. Experimental results reveal that the novel calibration method has an error compensation effect in the area where the intersection angle is close to the optimal junction angle and the measurement distance is not far, and that the error compensation effect is better in the area closer to the optimal measurement area. The results clearly demonstrate that the novel calibration method could improve the measurement accuracy of the wMPS, especially at the edge of the laser surface within a certain measurement area. This will broaden and deepen the fields of the wMPS application.

With the expanded high-accuracy measuring volume of the transmitter, the wMPS will be able to measure larger objects while it still keeps a high accuracy. The enlarged measurement volume also gives a hand to the improvement of the dynamic measurement accuracy of this system. However, this method still has certain limitations, that is, it has an error compensation effect within a certain measurement area. In future research, we will try to realize the error compensation in the areas of the poor intersection angle and the long measurement distance, or in this case, find out the main error source and design a new error compensation method, which can be used together with the method proposed in this paper.

## Figures and Tables

**Table 1:** The initial intrinsic parameters of the two transmitters.

Transmitter	a_1_	b_1_	c_1_	d_1_	a_2_	b_2_	c_2_	d_2_
1	0	−0.695745	0.718289	0	−0.779335	−0.002487	0.626602	−0.119252
2	0	−0.691038	0.722818	0	−0.695678	−0.010581	0.718275	1.32968

**Table 2:** The database of intrinsic parameters for the transmitter 1.

Region	a_11_	b_11_	c_11_	d_11_	a_12_	b_12_	c_12_	d_12_
$\beta <-12^{\circ }$	0	−0.695673	0.718358	0	−0.779260	−0.002488	0.626696	−0.119247
$-12^{\circ }\leq \beta <-4^{\circ }$	0	−0.695709	0.718324	0	−0.779297	−0.002485	0.626649	−0.119249
$-4^{\circ }\leq \beta \leq 4^{\circ }$	0	−0.695757	0.718277	0	−0.779341	−0.002490	0.626599	−0.119251
$4^{\circ }<\beta \leq 12^{\circ }$	0	−0.695781	0.718254	0	−0.779373	−0.002491	0.626555	−0.119254
$\beta >12^{\circ }$	0	−0.695817	0.718219	0	−0.779411	−0.002487	0.626508	−0.119257

**Table 3:** The database of intrinsic parameters for the transmitter 2.

Region	a_21_	b_21_	c_21_	d_21_	a_22_	b_22_	c_22_	d_22_
$\beta <-12^{\circ }$	0	−0.691111	0.722749	0	−0.695606	−0.010580	0.718345	1.329637
$-12^{\circ }\leq \beta <-4^{\circ }$	0	−0.691074	0.722783	0	−0.695642	−0.010586	0.718310	1.329663
$-4^{\circ }\leq \beta \leq 4^{\circ }$	0	−0.691024	0.722832	0	−0.695687	−0.010584	0.718266	1.329661
$4^{\circ }<\beta \leq 12^{\circ }$	0	−0.691002	0.722852	0	−0.695714	−0.010578	0.718241	1.329710
$\beta >12^{\circ }$	0	−0.690964	0.722887	0	−0.695749	−0.010582	0.718205	1.329731
